# A Gigantic Congenital Right Atrial Appendage Aneurysm in an Infant: Ten-Year Follow-Up

**DOI:** 10.3390/children9101552

**Published:** 2022-10-13

**Authors:** Antonios Belegrinos, Aikaterini Giannakopoulou, Melina Nikolakea, Evaggelos Karanasios, Sophie Mavrogeni, George Markousis-Mavrogenis

**Affiliations:** 1 Faculty of Medicine, National and Kapodistrian University of Athens, Mikras Asias 75, 11527 Athens, Greece; 2 Cardiology Clinic, “Aghia Sophia” Children’s Hospital, Thivon and Papadiamadopoulou, 11527 Athens, Greece; 3 Onassis Cardiac Surgery Center, Leoforou Andrea Siggrou 356, Kallithea, 17674 Athens, Greece

**Keywords:** gigantic aneurysm, right atrial appendage, infant, cardiac imaging, follow up, right atrial appendage aneurism, RAAA, congenital renal disease

## Abstract

A gigantic right atrial appendage aneurysm (RAAA) is a rare condition usually discovered during the third decade of life after being symptomatic. We present an asymptomatic RAAA discovered early during the basic screening of an infant and its natural history, and a ten-year follow-up due to its parents being against operation.

## 1. Clinical Presentation

A 10-month-old male infant with no significant family history was referred for cardiological assessment due to a heart murmur, 2/6 on the Levine scale, discovered incidentally prior to planned partial nephrectomy due to right upper renal atrophy with an ipsilateral duplicated collecting system. The ensuing physical examination yielded no additional findings. The 12-lead electrocardiogram (ECG) revealed sinus rhythm and a right bundle branch block, and a subsequent radiograph showed a normal cardiothoracic ratio and normal pulmonary vascularity.

**Figure 1:**
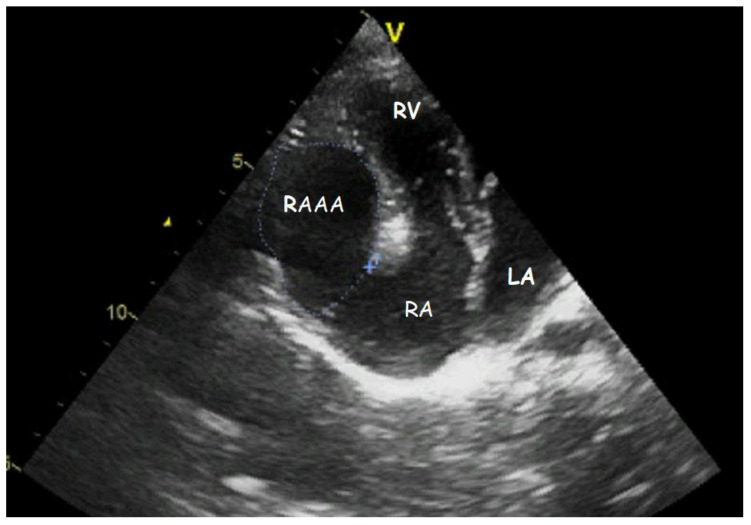
Transthoracic echocardiogram. Modified four−chamber view showing the gigantic RAAA.

**Figure 2:**
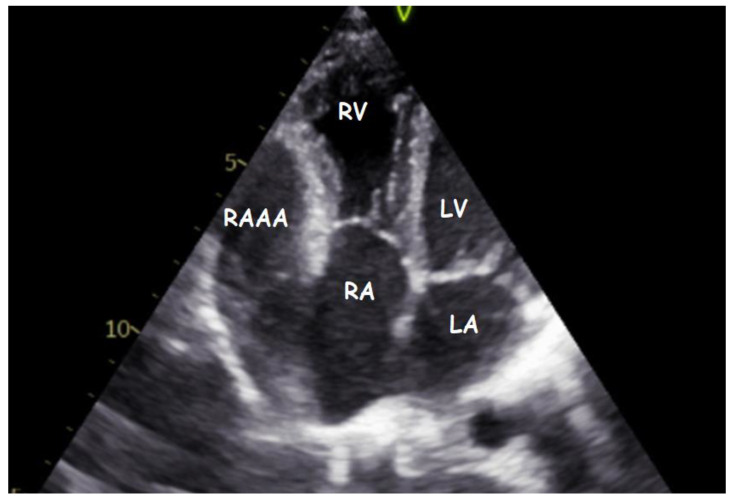
Transthoracic echocardiogram. Modified four-chamber view showing the gigantic RAAA ten years later.

**Figure 3:**
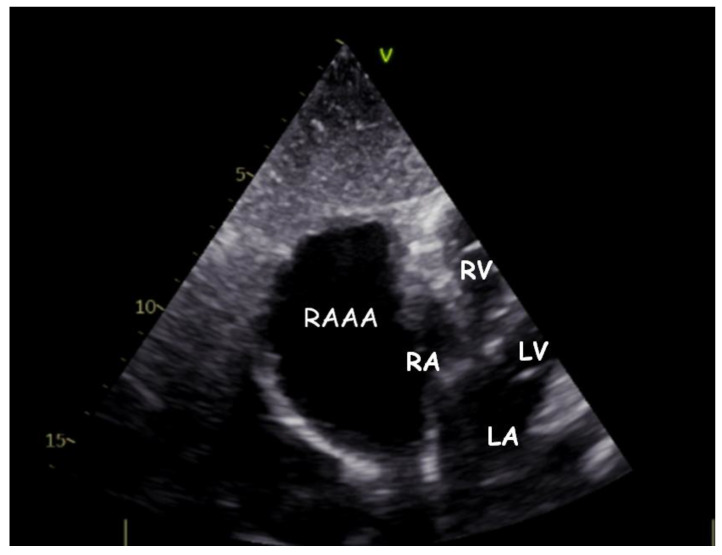
Transthoracic echocardiogram. Modified subcostal view showing the gigantic RAAA ten years later.

**Figure 4:**
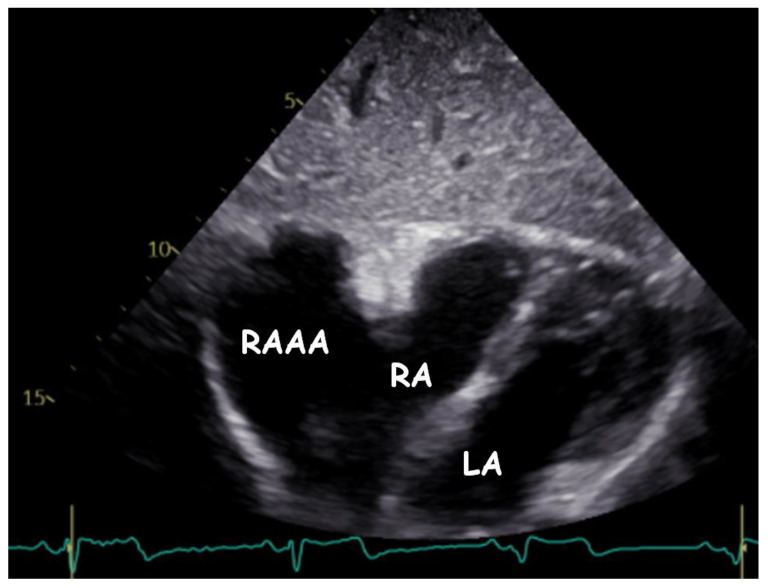
Transthoracic echocardiogram. Modified subcostal view showing the gigantic RAAA ten years later.

**Figure 5:**
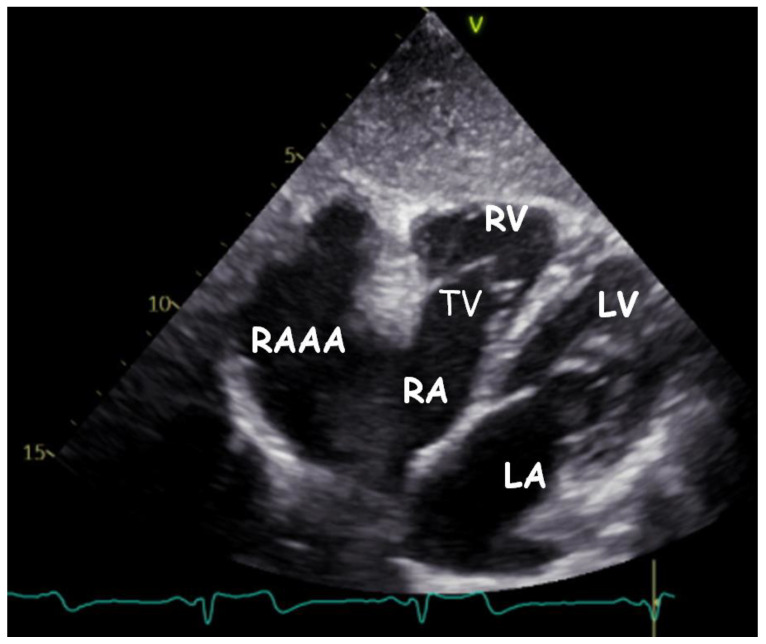
Transthoracic echocardiogram. Modified subcostal view showing the gigantic RAAA ten years later.

**Figure 6:**
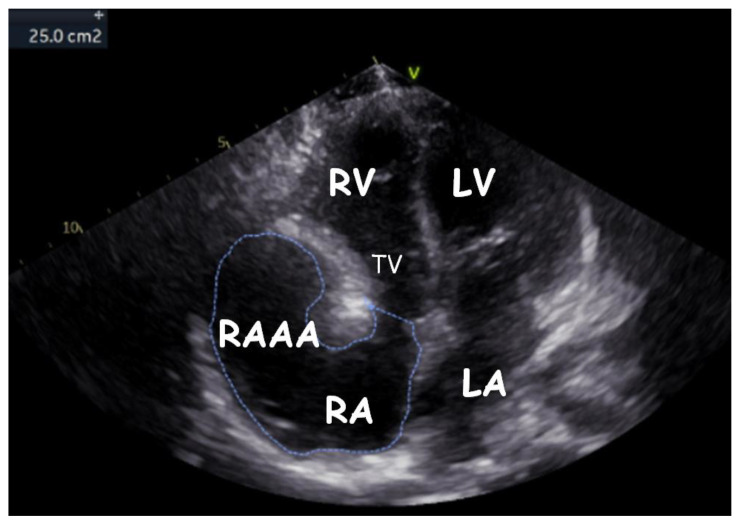
Transthoracic echocardiogram ten years later. Modified subcostal view showing the gigantic RAAA and the RA (estimated surface or RAAA + RA = 25 cm^2^).

**Figure 7:**
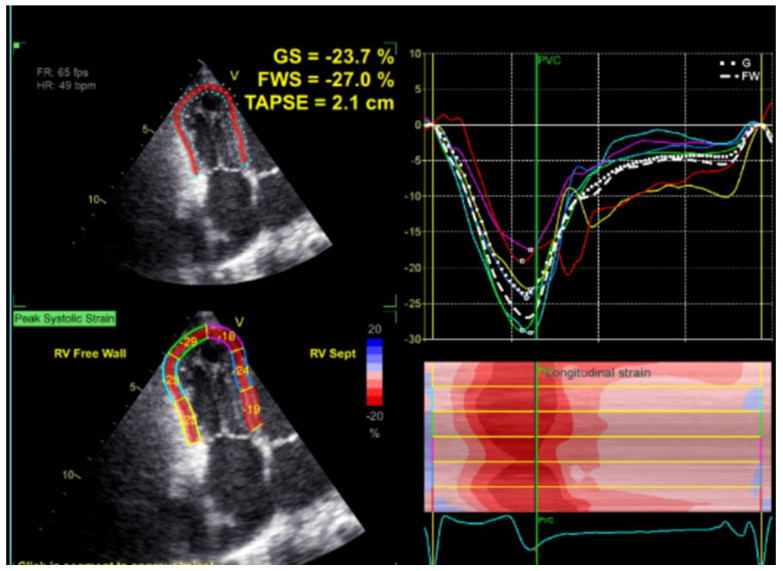
Normal stain of the right ventricle ten years later.

Transthoracic echocardiography (TTE) (Figure 1, Figure 2, Figure 3, Figure 4, Figure 5, Figure 6 and Figure 7, supplementary video available) revealed a giant right atrial appendage aneurysm (RAAA) that slightly compressed the basal right ventricular (RV) wall. The RAAA had a large communication with the RA and was also contracting with it. The diameter of the RV was 17 mm (normal range for body surface area = 0.46 m^2^ ≤ 18) and the left ventricle was in the normal range. No thrombus was present within the aneurysm. Both ventricles had normal contractility and blood flow through the tricuspid valve was not affected. The remaining echocardiographic characteristics were within normal ranges for his age.

**Figure 8:**
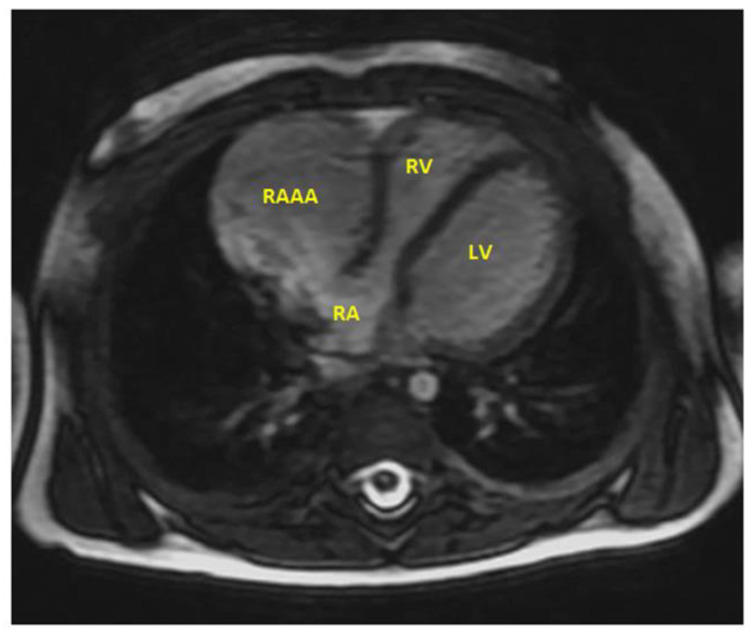
MRI in a ten-month-old infant. A gigantic aneurismal dilatation of the RAAA was detected.

**Figure 9:**
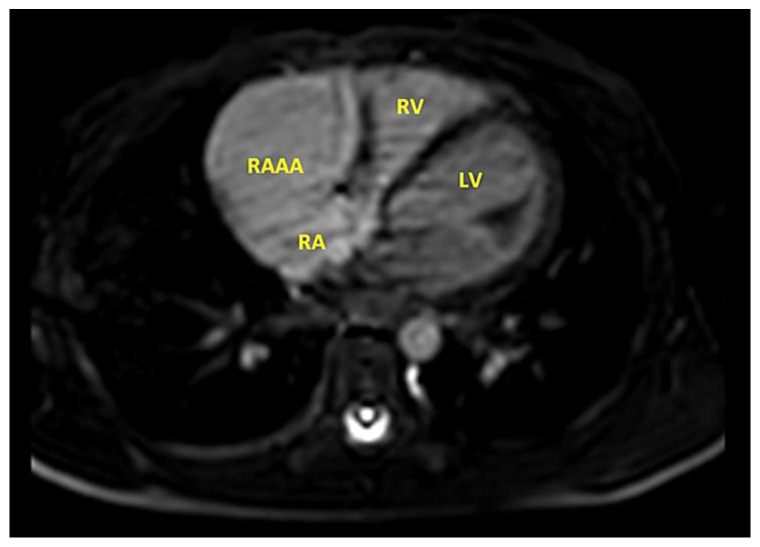
MRI in the same child eight years later. The initial gigantic aneurismal dilatation of the RAAA was evaluated again.

**Figure 10:**
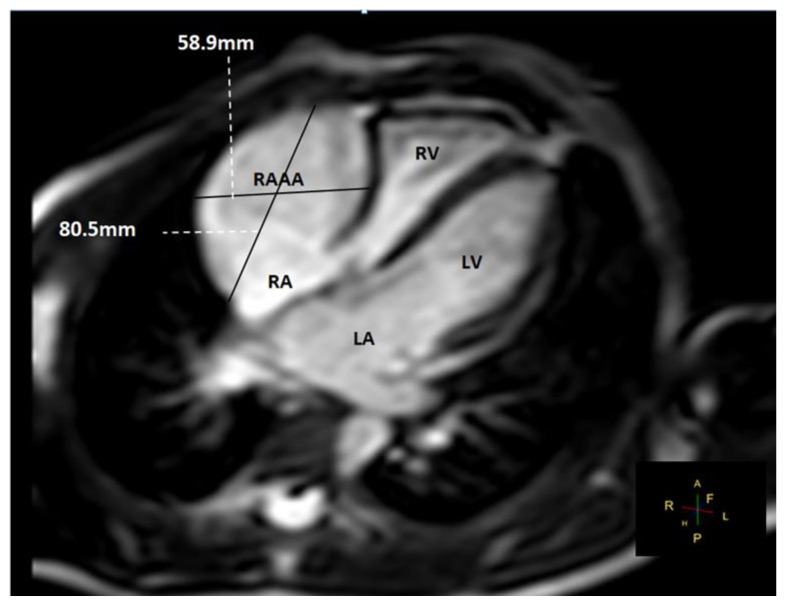
MRI in the same child eight years later. The initial gigantic aneurismal dilatation of the RAAA had diameters of 80 mm × 59 mm, reaching a total volume of 110 mL.

Magnetic resonance imaging (MRI) (Figure 8, Figure 9 and Figure 10) was performed to better visualize the cardiac structure and confirmed the aforementioned results. The size of the RAAA was 39 mm × 33 mm. The neck of the RAAA had a diameter of 27 mm at the end-diastole and 15 mm at the end-systole. It also revealed that the aneurysm was thick-walled. The right coronary was not compressed by the RAAA at rest, despite the two being in contact for 25 mm.

In the next MRI follow-up, seven years later, the aneurysm continued to grow, reaching a total size of 80 mm × 59 mm and a total volume of 110 mL.

## 2. Management

The heart team suggested surgical resection and repair. The patient’s parents declined surgical treatment. Acetylsalicylic acid (aspirin) was administered at 5 mg/kg per day to prevent thrombus formation. Strenuous physical activity was strongly discouraged. A close follow-up was performed every 6 to 12 months with TTE and 24 h ambulatory ECG monitoring in order to prevent complications. Arrhythmia was not detected during the evaluations and no thrombus formation was ever found in TTE and in sporadic MRIs.

## 3. Discussion

RAAA is a very rare condition, especially in infants, characterized by the dilatation of the right atrial appendage (RAA) [1,2]. The majority of cases are congenital with unknown etiology and, until now, no concrete genetic predisposition has been established [3]. Suggested mechanisms that could facilitate the creation of an RAAA without the existence of other structural abnormalities and without elevated right atrial pressure include defects of structural proteins, dysplastic pectinate muscles, and an abnormal collagen structure [4]. Owing to the possibility of a genetic link between renal birth defects and congenital heart diseases, with some studies demonstrating 30% co-occurrence [5], we support screening patients with congenital renal disease for possible congenital heart anomalies. Our search of the PubMed database did not yield any case reports of infants with RAAA and congenital kidney disease, with this case being perhaps one of the first reported. Most cases are diagnosed during adulthood, commonly during the third decade of life [1]. Sporadic cases have been documented from newborns to the eighth decade of life. Most patients with RAAA are asymptomatic and are often identified incidentally during cardiac imaging evaluation [2]. Dyspnea, palpitations, and/or thromboembolic phenomena are the most common clinical presentations [1]. Overt heart failure is rare [4]. Possible complications in RAAA include arrhythmias (most commonly atrial tachyarrhythmias, atrial flutter, and atrial fibrillation [6]), thromboembolic events, and rupture of the wall of the aneurysm [7,8,9]. Surgical resection seems to cure arrhythmias caused by RAAAs [6,10,11]. One article stated that, in four patients with similar pathology, drug treatment had either no effect or was only partially effective [6]. Furthermore, it has been stated that surgical resection could abolish the original A.T. [12]. Two of the patients studied reverted to A.T., which was successfully controlled by using antiarrhythmic medication [6]. Radiofrequency ablation is considered to have poor efficacy [11].

Of note is the fact that it is possible to detect RAAA in fetuses through echocardiography using a four-chamber view and the view of the right ventricular inflow tract [12]. Some atrial tachyarrhythmias may persist after birth [12]. In one case report, the role of transesophageal echocardiography was considered to be very important because of the quality of information provided to the surgical team that operated on that patient [13].

Some investigators suggest that surgical resection is indicated in symptomatic patients with RAAA [1]. Even though there is no consensus regarding treatment, in this particular case, because of its huge size and the possibility of complications, surgical repair and resection of the aneurysm are suggested.

Even though there is no consensus on the pharmaceutical treatment of these types of cases, due to the size, the possibility of low flow inside the aneurism, and the danger of suddenly developing atrial fibrillation, we suggest aggressive treatment with appropriate anticoagulation in order to avoid thromboembolic disease.

It was attempted to convince the patient and his parents to restrict exercise, as there are no extensive guidelines on the subject and it is unknown if the aneurismatic wall of the RAA would be damaged under the strain of intense exercise. Another concern was that. during high-intensity exercise, more so in contact sports, a significant strike to the chest area could theoretically cause a rupture of the aneurism.

One point of interest is that the patient was well for an extensive period of time, despite not following the conventional approach that states that such aneurisms must be operated upon. We are of the opinion that, until concrete, evidence-based guidelines have been established, a personalized approach should be followed. If the patient is symptomatic and able to undergo surgery, as discussed above, the general consensus is surgical resection and repair. Should the patient be completely asymptomatic, then perhaps the surgery could be postponed to a later date, given close follow-ups. We believe that this case, while being one singular observation, could point toward possible gaps in our knowledge and treatment of similar pathologies. Perhaps the formation of a national or international registry of all cases of RAAs in children, alongside complications that arose and our interventions, surgical or not, could lead to a better understanding of the disease’s natural history and clear guidelines that could help us to decide the best course of action and discern possible surgical indications.

## 4. Follow-Up

After ten years, the boy remains asymptomatic, without any complications, and shows compliance with the pharmaceutical treatment. There was a progressive increase in the size of the aneurysm and a mild right ventricular compression without signs of heart failure, intracardial thrombus formation, or thromboembolism. Against our suggestions, the child’s parents allowed moderate exercise, which turned out to be well-tolerated by the patient. The parents eventually agreed to surgery given the continual growth of the aneurism, and the child was referred for surgical repair.

## 5. Conclusions

RAAA can be found through careful TTE examination, even though it is not the gold standard for diagnosing this pathology. Surgery is advised, but if the patient is unwilling or unable to undergo surgical resection, close follow-ups, thrombosis prophylaxis, and other appropriate pharmaceutical interventions are needed in order to attempt to minimize the chance of a life-threatening complication occurring. Further research should be conducted on the subject, especially in cases where the patient is asymptomatic.

## 6. Learning Objectives

1. RAAA can remain asymptomatic for a long period of time, especially in children.
2. MRI provides useful information regarding the cardiac structure, the thickness of the wall, and the route of the right coronary artery, as well as potential sites of compression.
3. In patients who have not undergone surgery, close follow-ups and treatment are needed in order to avoid life-threatening complications.
4. There is a need for a national or international registry of cases of RAAA in children in order to better understand the natural history of the disease and create guidelines that help us handle similar cases.

## Supplementary Materials

The following supporting information can be downloaded at: https://www.mdpi.com/article/10.3390/children9101552/s1, Video S1: RAAA modified four-chamber view; Video S2: RAAA modified four-chamber view showing blood flow within the appendage.

## Abbreviations

RAAA	Right Atrial Appendage Aneurysm
RAA	Right Atrial Appendage
RV	Right Ventricle
RA	Right Atrium
LV	Left Ventricle
LA	Left Atrium
TTE	Transthoracic Echocardiogram
ECG	Electrocardiogram
MRI	Magnetic Resonance Imaging
A.T.	Atrial Tachyarrhythmia
TV	Tricuspid Valve
TAPSE	Tricuspid Annular Plane Systolic Excursion
GS	Global Strain
FWS	Free Wall Strain
